# Staff attitudes and the associations with treatment organisation, clinical practices and outcomes in opioid maintenance treatment

**DOI:** 10.1186/1472-6963-10-194

**Published:** 2010-07-06

**Authors:** Linn Gjersing, Helge Waal, John RM Caplehorn, Michael Gossop, Thomas Clausen

**Affiliations:** 1 SERAF- Norwegian Centre for Addiction Research, Institute of Clinical Medicine, University of Oslo, (Kirkeveien 166), Oslo, (0407), Norway; 2 Sydney School of Public Health, University of Sydney, (Edward Ford Building A27), NSW, (2006), Australia; 3 Kings College London, National Addiction Centre/Institute of Psychiatry, (4 Windsor Walk), London (SE5 8AF), UK

## Abstract

**Background:**

In opioid maintenance treatment (OMT) there are documented treatment differences both between countries and between OMT programmes. Some of these differences have been associated with staff attitudes. The aim of this study was to 1) assess if there were differences in staff attitudes within a national OMT programme, and 2) investigate the associations of staff attitudes with treatment organisation, clinical practices and outcomes.

**Methods:**

This study was a cross-sectional multicentre study. Norwegian OMT staff (*n *= 140) were invited to participate in this study in 2007 using an instrument measuring attitudes towards OMT. The OMT programme comprised 14 regional centres. Data describing treatment organisation, clinical practices and patient outcomes in these centres were extracted from the annual OMT programme assessment 2007. Centres were divided into three groups based upon mean attitudinal scores and labelled; "rehabilitation-oriented", "harm reduction-oriented" and "intermediate" centres.

**Results:**

All invited staff (*n *= 140) participated. Staff attitudes differed between the centres. "Rehabilitation-oriented" centres had smaller caseloads, more frequent urine drug screening and increased case management (interdisciplinary meetings). In addition these centres had less drug use and more social rehabilitation among their patients in terms of long-term living arrangements, unemployment, and social security benefits as main income. "Intermediate" centres had the lowest treatment termination rate.

**Conclusions:**

This study identified marked variations in staff attitudes between the regional centres within a national OMT programme. These variations were associated with measurable differences in caseload, intensity of case management and patient outcomes.

## Background

Opioid maintenance treatment (OMT) is recognised as an effective treatment in opiate dependence [1-3]. However OMT is a contentious issue and there are many views on how to organise this treatment [4,5]. OMT programmes differ in treatment objectives, organisation and clinical practices [6,7]. Treatment objectives range from harm reduction [8], long-term maintenance and rehabilitation [2], to abstinence from all drugs including treatment medication [9]. Diversity in treatment is evident between countries [10], within countries [11], and even between counsellors within the same treatment programme [12]. Such differences have been associated with variations in programme policies and staff attitudes [13-15].

Several studies have assessed staff attitudes in the addiction field [16-19]. Differences in staff attitudes have been associated with treatment practices and outcomes [20-22]. OMT staff's favour towards abstinence-models has been associated with provision of low dose methadone [22,23]. Abstinence-oriented OMT staff have had more in-treatment drug use and more drop out compared to programmes where staff were long-term maintenance-oriented [20,21,24]. Strong attitudes and the beliefs of the majority are more likely to influence behaviour than weaker attitudes held by only one or few persons [25-28]. Consequently staff attitudes should be included when OMT programmes are assessed.

There are between 8 200 and 12 000 injecting drug users in Norway [29] and approximately 5000 are currently in OMT [30]. In Norway OMT is only available through a single publicly funded programme [31]. The programme was established in 1998 and it has had a rapid expansion from the initial intake of 240 patients to 4542 patients in 2007 [30]. The programme comprises 14 regional centres that are subject to the same treatment standards specified in government guidelines [31].

Annual assessments of the OMT programme indicate marked differences in treatment organisation, practices and outcomes between centres [32,33]. This is of concern since not all centres appear to achieve outcomes in line with specified programme aims; reduced drug use and improved social rehabilitation. Also OMT in Norway relies on long-term three-party collaboration between an OMT centre, a GP and social services, thus patients are not entirely free to choose their treatment centre due to logistical and geographical challenges. This means that patients are required to accept their local centre's treatment standards and practices. Thus it is important to investigate factors that may be associated with differences in treatment delivery and outcomes. The aim of this study was to 1) assess if there were differences in staff attitudes between OMT centres, and 2) investigate the associations of staff attitudes with treatment organisation, clinical practices and outcomes.

## Methods

### Setting

All clinical OMT staff (*n *= 140) in full-time and part-time positions, in the national OMT programme in Norway were invited to participate in this study. A list of all clinical staff at each centre ensured that all staff were invited. The national OMT programme comprised of fourteen centres, and had from three to thirty-three staff employed. In this study two of the fourteen centres were merged because they had a joint staff group at the time of the study.

### Design

The study was a cross-sectional multicentre study. Data was collected from August to November 2007, through visits by the first author. The visits were in conjunction with staff meetings when most staff were present. Staff that were absent returned the questionnaire by mail. The first author was present during the completion of the questionnaires in all except one OMT centre. In the latter OMT centre the researcher gave information during a staff meeting and thereafter questionnaires were returned by mail. Prepaid and anonymous envelopes addressed to the researcher were attached to each questionnaire. Responders could choose not to respond to the survey by returning an incomplete questionnaire in the envelope. No names were collected, but number of staff that had completed the questionnaire at each facility was recorded. Centre managers were followed up to encourage that all staff returned questionnaires.

The study was approved by the Norwegian Regional Ethics Committee and the Data Inspectorate May-June 2007. Participants received written and oral information about the study. Respondents consented to participate in the study by submitting the questionnaire. The questionnaire was semi-anonymous. This means that the name of the facility and other demographic variables made some staff theoretically identifiable. Participants were promised full anonymity. Demographic variables that identify respondents will therefore be deleted upon completion of the project.

### Study instrument

The study instrument included a 13-item attitudinal scale (Table 1). This scale was developed through exploratory factor analysis and confirmed in structural equation modelling using maximum likelihood analysis [34]. The scale comprised two factors; "compliance" and "accessibility" that were highly correlated in this OMT sample (r = 0.71) [34]. The "Compliance"-items measured attitudes towards in-treatment drug use in OMT and the "accessibility"-items measured attitudes towards who should have access to an OMT programme. The development of the scale is described in details elsewhere [34]. Participants were asked to rate their responses to each item on a five-point Likert scale from strongly disagree = 1 to strongly agree = 5. Total scores were divided by number of questions answered [35]. The theoretical range of mean scores was 1.00 to 5.00.

**Table 1:** The 13-item scale measuring attitudes towards opioid maintenance treatment*

1	OMT patients who ignore repeated warnings to stop using heroin should be gradually withdrawn off methadone
2	OMT patients who continue to abuse non-opioid drugs (e.g. benzodiazepines) should have their dose of OMT medication reduced.

3	If repeated warnings of non-prescriptive use of benzodiazepines are ignored, the patient should be discharged from the OMT programme

4	If repeated warnings of use of Cannabis are ignored, the patient should be discharged from treatment (OMT)

5	The GP should waive the right to prescribe class A and B drugs other than the OMT medication to OMT patients

6	OMT patients who continue to take drugs and function poorly should be discharged from the OMT programme

7	It is unethical to discharge patients from the OMT programme due to continuing drug use and poor functioning**

8	OMT services should be expanded so all heroin addicts who want OMT can receive it**

9	It is unethical to deny heroin addicts OMT**

10	OMT's main aim is to reduce harmful effects of opioids and IV drug use (syringes)**

11	GPs should be able to initiate OMT on their own initiative**

12	Too many OMT-patients are discharged from the OMT programme**

13	Young opioid dependents (< 20) should not be offered OMT

*Participants were asked to rate their responses to each item on a five-point Likert scale from strongly disagree = 1 to strongly agree = 5.

** Reversed scores

Demographic variables such as treatment centre, age, gender, profession, time employed in the organisation and time worked in the addiction field were collected.

### Labelling

In this study it was decided to label those with the lowest mean scores on the attitudinal scale "harm reduction-oriented" and those with the highest mean scores "rehabilitation-oriented". These labels were based upon the content of the attitudinal scale. This means that the "harm reduction-oriented" would be more likely to disagree that drug use was a reason for disciplinary discharge, more likely to agree that an OMT programme should be available to all opiate dependents and more likely to agree that GPs should be able to treat OMT patients independently of the OMT programme. "Rehabilitation-oriented" would have opposite attitudes.

### Division of centres into attitudinal groups

Each centre's mean attitudinal score was assessed. Centres were divided into three groups based upon these scores, with equal number of centres in each group (4-5-4). The four centres with the lowest scores were termed "harm reduction-oriented" and the four centres with the highest scores were termed "rehabilitation-oriented". The five centres that had attitudinal scores between the two opposing groups were termed "intermediate" centres.

### OMT centre characteristics

The Norwegian OMT programme is assessed annually as part of an ongoing quality assessment. A 53-item questionnaire is to be completed for each OMT patient. The questionnaire comprises items such as main income, employment status and drug use previous four weeks. The questionnaire is completed by the patients' case manager. The questionnaire was reliability tested in 2005 [36] and revised according to findings. Data was collected as a multicentre study; however only aggregated information was available for analysis. This study used data collected in the third quarter of 2007.

### Treatment variables and treatment outcomes

Patient/staff ratio, methadone and buprenorphine dose, and interdisciplinary meeting attendance among patients as well as supervised dispensing and urine drug screening at each OMT centre were selected as treatment variables. Drug use and social functioning variables were outcome variables. Drug use variables were opioid, benzodiazepines, central stimulants and cannabis use previous four weeks. These data were measured by urine testing and self-report. Social functioning was measured using current employment status, social security benefits as main income and type of living arrangements. Patient retention was measured indirectly using the treatment termination rate. This rate was calculated by adding all patients at the beginning of the year to all new patients throughout the year (n_1_). Thereafter this (n_1_) was subtracted from the total number of patients at the end of the year (n_2_). Finally this (n_1_-n_2_) was divided by the sum of all patients at the beginning of the year and all new patients throughout the year (n_1_).

### Data analysis

Descriptive statistics and regression analysis were completed using SPSS version 16.0 [37]. A missing value pattern was generated for all items. Staff attitudes were investigated by linear regression analysis with mean attitudinal score as dependent variable and age, gender, staff category, years of education, time worked in the addiction field and OMT centre as independent or predictor variables. Prevalence estimates were reported [38]. Differences between centres were calculated using prevalence difference and 95% CI. Data on centre characteristics were only available as aggregated information (number of patients for each variable and total number of patients) for each regional centre (14 centres) was available for analysis. Only completed items, from the annual OMT assessment, were included in the analysis. Not all items in each patient questionnaire were completed, thus the total number of respondents for each item varied from the total number of patients at each centre.

## Results

### Respondents

All invited staff (*n *= 140) responded. One questionnaire (1%) was discarded due to incomplete answers. Two questionnaires had two missing responses and five questionnaires had one missing response, all items were completed in the remaining questionnaires. There were no observed differences in age, gender, occupation and length of employment in the addiction field between OMT centres. 63% of staff were women. 59% of staff were either social workers or registered nurses, and 21% were psychologists and doctors. Other staff categories were teachers and social educators. The majority of staff (60%) were between forty and fifty-nine years old. No staff were below twenty-five years. 62% had worked in the addiction field more than six years.

### OMT centres and staff attitudes

OMT centre was the only independent variable that was associated with staff attitudes (r = 0.44, b = 0.06 95% CI 0.04; 0.08). No other personal descriptors contributed to explaining variations in staff attitudes. An assessment of each OMT centre found that mean attitudinal scores varied from 2.50 (95% CI 2.01; 2.99) to 4.26 (95% 4.02; 4.51).

### Treatment practices and staff attitudes

There were 4542 patients in the OMT programme by the end of 2007. The four centres with the lowest attitudinal scores ("harm reduction-oriented") comprised 1980 patients. The four centres with the highest attitudinal scores ("rehabilitation-oriented") comprised 1049 patients (Table 2). Centres between the two opposing groups in attitudinal scores ("intermediate") comprised 1513 patients. The patient/staff ratio varied between groups. The "harm reduction-oriented" centres had a much higher patient/staff ratio (30% (95% CI 27%; 34%, p > 0.001) than the "rehabilitation-oriented" centres (Table 2).

**Table 2:** Treatment characteristics and practices for OMT centres when divided into attitudinal groups

Characteristics for each group	"Harm reduction-oriented" centres	"Intermediate" centres	"Rehabilitation-oriented" centres
Total number of patients per group	1980	1513	1049

Patient/staff ratio	64	50	34

Methadone dose (mg)*	111 mg	111 mg	106 mg

Buprenorphine dose (mg)*	18 mg	17 mg	20 mg

Number of supervised dispensing per patient per week*	4.1	3.9	3.7

Number of patients urine drug screened at least once a week *†	62% *(1419)*	73% *(1325)*	74% *(1115)*

Interdisciplinary meeting previous 4 weeks*†	46% *(1493)*	49% *(1326)*	59% *(1070)*

*Median

† Number of patients in brackets

†† Not all items in each patient questionnaire were completed, thus the total number of respondents for each item varied from the total number of patients at each centre.

All three groups had a high dose policy. The median methadone dose varied from 106-111 mg and buprenorphine from 17-20 mg between groups (Table 2). Number of supervised dispensing per patient per week, i.e. the use of "take-home doses" did not differ greatly between groups. However "harm reduction-oriented" centres were 12% (95% CI 5%; 18%, p > 0.001) less likely to collect weekly urine specimens than the "rehabilitation-oriented". In addition there were differences in number of patients attending interdisciplinary meetings. 13% less patients (95% CI 8%; 19%, p > 0.001) attended interdisciplinary meetings the previous four weeks in the "harm reduction-oriented" centres compared to the "rehabilitation-oriented".

The "intermediate" centres had rates in treatment characteristics and practices between the "harm reduction-oriented" and "rehabilitation-oriented" in most variables (Table 2).

### Treatment outcomes and staff attitudes

"Harm reduction-oriented" centres had more in-treatment drug use compared to the two other groups (Table 3). More than half of the patients (55%) had used benzodiazepines previous four weeks compared to 32% in "rehabilitation-oriented" centres. Furthermore "harm reduction-oriented" centres had less social functioning among their patients compared to the "rehabilitation-oriented" centres; 17% more patients were unemployed, 12% more patients had social security benefits as main income and 15% less patients had long-term living arrangements (Table 3). The "intermediate" centres were between the "harm reduction-oriented" and "rehabilitation-oriented" centres in all treatment outcomes. The exception was the treatment termination rate, where the "intermediate" centres had the lowest rate.

**Table 3:** Treatment outcomes for OMT centres when divided into attitudinal groups

Treatment outcomes for each group	"Harm reduction-oriented" centres	"Intermediate" centres	"Rehabilitation-oriented" centres	Prevalence difference* (95% CI)	p-value**
Opioids use previous 4 week	18% *(1260)*††	15% *(1215)*	14% *(1092)*	4% (1%; 7%)	0.022

Central stimulant drug use previous 4 week	19% *(1227)*	19% *(1200)*	16% *(1093)*	3% (-1%; 6%)	0.102

Benzodiazepine use previous 4 weeks	55% *(1296)*	50% *(1229)*	32% *(1102)*	23% (18%; 29%)	< 0.001

Cannabis use previous 4 weeks	46% *(1258)*	41% *(1224)*	21% *(1090)*	25% (20%; 29%)	< 0.001

Unemployed in treatment	83% *(1503)*	77% *(1342)*	66% *(1112)*	17% (10%; 23%)	< 0.001

Social security benefits as main income	24% *(1494)*	15% *(1257)*	12% *(1111)*	12% (9%; 15%)	< 0.001

Long-term living arrangements for patients in treatment	71% *(1503)*	82% *(1344)*	86% *(1116)*	-15% (-21%; -8%)	< 0.001

Treatment termination rate	11% *(2213)*	9%† *(1667)*	15% *(1241)*	- 4% (-8%; - 2%)	< 0.001

Total number of patients (n) per item in brackets

*Prevalence difference in percent. It was calculated by subtracting the prevalence of "harm reduction-oriented" centres from the prevalence of the "rehabilitation-oriented" centres.

**P-values estimated for prevalence difference

† Prevalence difference ("rehabilitation-oriented" vs. "intermediate" centres) 5% (4%; 9%), p-value < 0.001)

††Not all items in each patient questionnaire were completed, thus the total number of respondents for each item varied from the total number of patients at each centre.

## Discussion

Within one national OMT programme there were differences between the regional centres in staff attitudes and these differences were associated with variations in treatment organisation, clinical practices and outcomes. Staff in "rehabilitation-oriented" centres were more likely to agree that drug use was a reason for disciplinary discharge, disagree that an OMT programme should be available to all opiate dependents and less likely to agree that GPs should be able to treat OMT patients independently of the national OMT programme. "Rehabilitation-oriented" centres had smaller caseloads, more frequent urine drug screening and increased case management (interdisciplinary meetings). In addition these centres had less drug use and more social rehabilitation among their patients in terms of long-term living arrangements, unemployment, and social security benefits as main income. "Intermediate" centres had the lowest treatment termination rate.

Treatment approach has been associated with staff attitudes [13]. Abstinence-oriented staff have provided lower methadone doses compared to long-term maintenance oriented staff [20-22]. Staff in pharmacies with negative attitudes towards drug users have been less likely to provide needle exchange services [39]. Psychiatric staff with positive attitudes towards seclusion of patients have been more likely to be involved in this treatment practice [40]. In the current study centres with "rehabilitation-oriented" staff urine drug screened more frequently and had increased case management (interdisciplinary meeting attendance) of their patients.

"Harm reduction-oriented" centres had almost double caseload compared to the "rehabilitation-oriented" centres. Staff in these centres were more likely to agree that OMT programmes should be open to all opiate dependents and possibly attempted to admit all those who presented for treatment. Conversely it is possible that staff in "rehabilitation-oriented" centres emphasised provision of services and limited their intake to ensure a manageable caseload.

Caseload affects services provided [41,42]. Smaller caseloads allow staff more time per patient and this affect the services provided [41-43]. However small caseloads are not sufficient to ensure beneficial treatment outcomes; quality of services, such as case management, are also important [2]. Several studies have demonstrated that increased case management are associated with less drug use and increased social rehabilitation [44,45]. The "rehabilitation-oriented" centres had smaller caseloads and more intense case management of their patients. This is possibly one of the reasons why they had less drug use and more social rehabilitation among their patients compared to the "harm reduction-oriented" centres.

Staff disapproval of in-treatment drug use could be another reason why "rehabilitation-oriented" centres had less drug use and more social rehabilitation among their patients. High expectations of functioning have been found to enhance patients' engagement in treatment services and subsequently treatment outcomes [43]. Staff in "rehabilitation-oriented" centres were more likely to agree that in-treatment drug use was a reason for discharge from treatment. It is possible that patients were motivated to abstain from drug use because staff disapproved of in-treatment drug use, and thus staff attitudes influenced patient's outcomes positively.

It is also possible that the "rehabilitation-oriented" centres only included patients that were less severely affected and with a higher level of social functioning. A differential selection of patients into treatment would possibly influence level of drug use and social functioning of patients in treatment. Baseline information on each patient was not available. However there was no difference between centres in patients' age and gender distribution. In addition the government regulations of the OMT programme specify that only those with long-term opioid dependence should be accepted into treatment. It is therefore unlikely that the patients' characteristics alone i.e. severity of dependence and drug use patterns, could explain all the observed variations in social rehabilitation and drug use.

Drug use is an important measure in OMT. Especially benzodiazepine use has been related to increased risk of overdose, other drug use, sharing injecting equipment, and more psychopathology and social dysfunction [46-48]. The "rehabilitation-oriented" centres had less drug use and higher rates of social rehabilitation among their patients.

Retention in treatment is another important treatment indicator as OMT protect patients against increased risk of mortality [49]. All centres had low treatment termination rates, but the "intermediate" centres had the lowest. It may be that too strong attitudes ("harm reduction-oriented" or "rehabilitation-oriented") in either directions influence termination rates in OMT negatively.

Overall the cross-sectional design prevented any inference as to causality. Differences in treatment outcomes were not necessarily caused by differences in either staff attitudes or centre policies. Aggregated information prevented detailed analysis based upon individual patients. In addition the disciplinary discharge of patients for continuing drug use would reduce the proportion of patients using drugs while in treatment. There were no systematic patterns between response rate and treatment practices and outcomes, which suggested that selection bias was not a major concern.

Despite these limitations, the present study provides a detailed description of the Norwegian OMT programme and differences in staff attitudes, centres and patient outcomes. This study was able to identify significant differences in staff attitudes, treatment practices and outcomes between OMT centres. Furthermore all eligible staff participated in this study which support the validity of the findings, as it was not only a selection of OMT staff that responded.

## Conclusions

This study identified marked variations in staff attitudes between the regional centres within one national OMT programme. These variations were associated with measurable differences in caseload, intensity of case management and patient outcomes. These findings add to the body of evidence that staff attitudes are associated with programme policies and the intensity and style of case management and, subsequently, patient outcomes in opioid maintenance treatment. Policy makers and stakeholders, as well as programme managers and OMT staff need to be aware of this.

## Competing interests

The authors declare that they have no competing interests.

## Authors' contributions

LG participated in the conception and design of the study, carried out the data collection, analyzed and interpreted the data and drafted the manuscript. HW participated in the conception and design of the study, interpreted the data and revised the manuscript critically for intellectual content. JRMC participated in the analysis and interpretation of the data and revised the manuscript critically for intellectual content. MG participated in the analysis and interpretation of the data and revised the manuscript critically for intellectual content. TC participated in the conception and design of the study, participated in the interpretation of the data and revised the manuscript critically for intellectual content. All authors read and approved the final manuscript.

## Pre-publication history

The pre-publication history for this paper can be accessed here:

http://www.biomedcentral.com/1472-6963/10/194/prepub
